# Small-area models to assess the geographical distribution of tobacco consumption by sex and age in Spain

**DOI:** 10.18332/tid/162379

**Published:** 2023-05-18

**Authors:** María I. Santiago-Pérez, Esther López-Vizcaíno, Mónica Pérez-Ríos, Carla Guerra-Tort, Julia Rey-Brandariz, Leonor Varela-Lema, Lucía Martín-Gisbert, Alberto Ruano-Ravina, Anna Schiaffino, Iñaki Galán, Cristina Candal-Pedreira, Agustín Montes, Jasjit Ahluwalia

**Affiliations:** 1 Epidemiology Department, Directorate-General of Public Health, Galician Regional Health Authority, Santiago de Compostela, Spain; 2 Diffusion and Information Service, Galician Institute of Statistics, Santiago de Compostela, Spain; 3 Department of Preventive Medicine and Public Health, University of Santiago de Compostela, Santiago de Compostela, Spain; 4 Consortium for Biomedical Research in Epidemiology and Public Health (CIBER en Epidemiología y Salud Pública/CIBERESP), Santiago de Compostela, Spain; 5 Health Research Institute of Santiago de Compostela (Instituto de Investigación Sanitaria de Santiago de Compostela - IDIS), Santiago de Compostela, Spain; 6 Directorate-General of Health Planning, Health Department, Catalonian Regional Authority, Barcelona, Spain; 7 National Centre for Epidemiology, Carlos III Institute of Health, Madrid, Spain; 8 Department of Preventive Medicine and Public Health, Autonomous University of Madrid/IdiPAZ, Madrid, Spain; 9 Department of Medicine, Alpert School of Medicine, Brown University, Providence, United States; 10 Department of Behavioral and Social Science, School of Public Health, Brown University, Providence, United States; 11 Legoretta Cancer Center, Division of Biology and Medicine, Brown University, Providence, United States

**Keywords:** small-area analysis, prevalence, smoking, health surveys, behavioral risk factor surveillance system

## Abstract

**INTRODUCTION:**

Complete and accurate data on smoking prevalence at a local level would enable health authorities to plan context-dependent smoking interventions. However, national health surveys do not generally provide direct estimates of smoking prevalence by sex and age groups at the subnational level. This study uses a small-area model-based methodology to obtain precise estimations of smoking prevalence by sex, age group and region, from a population-based survey.

**METHODS:**

The areas targeted for analysis consisted of 180 groups based on a combination of sex, age group (15–34, 35–54, 55–64, 65–74, and ≥75 years), and Autonomous Region. Data on tobacco use came from the 2017 Spanish National Health Survey (2017 SNHS). In each of the 180 groups, we estimated the prevalence of smokers (S), ex-smokers (ExS) and never smokers (NS), as well as their coefficients of variation (CV), using a weighted ratio estimator (direct estimator) and a multinomial logistic model with random area effects.

**RESULTS:**

When smoking prevalence was estimated using the small-area model, the precision of direct estimates improved; the CV of S and ExS decreased on average by 26%, and those of NS by 25%. The range of S prevalence was 11–46% in men and 4–37% in women, excluding the group aged ≥75 years.

**CONCLUSIONS:**

This study proposes a methodology for obtaining reliable estimates of smoking prevalence in groups or areas not covered in the survey design. The model applied is a good alternative for enhancing the precision of estimates at a detailed level, at a much lower cost than that involved in conducting large-scale surveys. This method could be easily integrated into routine data processing of population health surveys. Having such estimates directly after completing a health survey would help characterize the tobacco epidemic and/or any other risk factor more precisely.

## INTRODUCTION

Current global estimates are that 20% of the world population are smokers – 975 million men and 175 million women smoke. In recent decades, the number of smokers has risen, resulting in tobacco use continuing to be one of the most important risk factors worldwide^1^. There is a need for estimates of the prevalence of tobacco use at a subnational level, to improve surveillance, identify inequalities, and design and implement primary or secondary prevention interventions and effective context-dependent policies. Conversely, population-based surveys having the necessary power to make nationwide estimates by sex and age do not generate detailed risk profiles at a subnational level. In the case of risk factors that do not display homogeneous geographical prevalence, such as tobacco, it is essential to have these estimates by sex and age group at a subnational level.

Estimating smoking prevalence by applying small-area estimation methods is less time-consuming and cheaper than collecting survey data in detail for population groups at the subnational level, since many people would need to be interviewed in each group to produce precise estimates, if these estimations derive from population surveys. If accepted as valid, the figures obtained could be used to identify specific populations with high smoking prevalence. If this approach were regularly applied, the figures obtained could be used to identify populations with unchanging or worsening smoking prevalence, improving the commissioning of targeted services to lower prevalence. Understanding the geography or spatial pattern of health-related behaviors at a subnational level (i.e. how the exposure of interest is spatially distributed) is essential. Small-area estimations of major health determinants give us a precise picture of the distribution of risk factors, something that is essential when it comes to implementing policies targeted at curtailing major population risk factors such as smoking.

Data from the Spanish National Health Survey, with an annual sample size of approximately 23000, show that the prevalence of tobacco use varies widely across Spain’s administrative health areas known as Autonomous Regions (ARs). However, the survey design does not allow for the prevalence to be precisely estimated for each AR by sex and age group. If such territorial differences were indeed present, these would go unnoticed where only AR-level prevalence estimates by sex were calculated, thus preventing the implementation of tobacco-related policies targeting specific populations in specific areas.

Small-area estimation methods are a valid and cost-effective alternative to direct estimates derived from surveys aimed in obtaining the prevalence of behavioral risk factors in specific groups with small sample sizes^2-6^. The aim of this study was, therefore, to apply a small-area model-based methodology to obtain precise estimations of smoking prevalence by sex, age group and region, from a population-based survey.

## METHODS

The units of analysis in this study were 180 groups or areas defined on the basis of Spain’s territorial division into ARs, as well as sex and age groups in the five categories mainly used to assess the health impact of tobacco consumption (15–34, 35–54, 55–64, 65–74, and ≥75 years), resulting 180 groups (18 ARs × 2 sexes × 5 age groups). We considered the country’s 17 ARs and the two Autonomous Cities of Ceuta and Melilla, considered as one region. In each group, we estimated the prevalence of smokers (S), ex-smokers (ExS) and never smokers (NS) in 2017, applying a small-area estimation (SAE) method that uses aggregate survey-based data on tobacco use and auxiliary information at an area level, sourced from administrative records. In the SAE methodology, the term small area, or area hereafter, refers to a group with a small sample size and not necessarily a geographical area.

### Data sources and study variables

Tobacco-use data were sourced from the 2017 Spanish National Health Survey (2017 SNHS), which targets the population aged ≥15 years residing in main family dwellings nationwide (n=23089). Data collection was performed by home-based computer-assisted personal interviewing (CAPI) from October 2016 through October 2017. The sample was selected by a stratified, three-stage sampling of census sections, households, and one adult per household, successively. SNHS-based estimates are representative of the population at an AR level, and an AR by sex is the smallest publicly available area. The SNHS includes several questions on smoking, which were used to create the categorical smoking variables of S, ExS and NS. Detailed information is provided elsewhere^7^.

By way of auxiliary data, we selected variables that were associated with tobacco use adapted to the context of the country under study and available for the 180 groups. Population data were sourced from the 2017 Population Register, and unless otherwise indicated, all variables are from 2017. The variables were as follows: 1) nationality – percentage of foreign population^8^; 2) degree of urbanization – population percentage living in densely populated local administrative units (DPA), intermediate populated local administrative units (IPA) and thinly populated local administrative units (TPA), as per the Eurostat Classification of Cities^8,9^; 3) population percentage living in coastal or inland towns and cities^9^; 4) educational level – population percentage with basic, secondary or higher education (2011 Census^10^); 5) relationship with activity –percentage of employed, unemployed or economically inactive population, and employment rate^11^; 6) main occupation – percentage of directors, managers, technicians and professionals, percentage of skilled and unskilled workers^11^; 7) occupational sector – percentage of employed population in industry, construction or services^11^; 8) income level – mean per capita income^12^, population percentage living in towns/cities with a deprivation index (DI)^13^ below the 10th percentile or above the 90th percentile (2011 Census); and 9) morbidity – population percentage hospitalized due to lung cancer and due to chronic obstructive pulmonary disease (COPD)^14^. In order to fit the model, quadratic variables were also considered. The data sources for each auxiliary variable are fully described in Supplementary file Table S1, and the download URL is also provided.

### Statistical analysis

Based on microdata sourced from the 2017 SNHS, available on the website of the National Statistics Institute (INE)^7^, we first calculated the prevalence of S, ExS and NS in the 180 groups, applying a weighted ratio estimator (direct estimate):


$$\hat{P}\;=\;\frac{\Sigma_{hi}W_{hi}X_{hi}}{\Sigma_{hi}W_{hi}}$$ (1)

where *h* indicates sample design stratum, *i* the individual, *X_hi_* is the value of the characteristic estimated (0–1) in individual *i* of stratum *h*, and *W_hi_* is the sampling weight of individual *i* in stratum *h*. The weighted ratio estimator of a proportion is a ratio between two total estimators: the total of the persons who have the characteristic (smoker, ex-smoker or never smoker in our study) and the total population. This estimator is what the National Institute of Statistics in Spain applies to obtain estimations from the SNHS. The variance of this estimator was calculated using a Taylor series linear approximation, and, based on this, the coefficients of variation (CV) were then obtained.

We identified a total of 6 areas in which the prevalence of smokers could not be estimated due to the absence of smokers in the sample: these areas corresponded to women aged ≥75 years in the Balearic Isles, Catalonia, Galicia, Murcia, La Rioja and Ceuta-Melilla. In the case of this last area, the prevalence of ex-smokers aged ≥75 years could also not be estimated.

The small-area estimation method is based on a multinomial logistic model with aggregated data by area and random area effects^15^. The dependent variable *Y* is the number of individuals in each area classified in *q* categories of a qualitative variable, which in this study is tobacco use and has 3 categories (S, ExS and NS). In this case, the last category is taken as reference, with the result that the *Y* vector is 2-dimensional (generally *q-1*).

To take the 2017 SNHS complex sample design into account, the number of smokers and ex-smokers in each area was calculated by multiplying the direct estimator of the relevant proportion by the total sample size of the area.

In each area *i*, the vector of sample totals *y_i_=(y_i1_,y_i2_)*' is assumed to follow a multinomial distribution conditional upon an area effect *u_i_=(u_i1_^,^u_i2_)':*


$$y_{i}|u_{i}∼M(n_{\text{i}},p_{\text{i}1},p_{\text{i}2}),i=1,2,\ldots ,\text{I}$$ (2)

where *I* is the number of areas, in this case 174; *p*_ik_=*Pr*(*y_ik_*=1|*u_i_*) is the likelihood of being a smoker (*k*=1) or ex-smoker (*k*=2); and the random effects u_i_ are independent and follow a normal distribution with mean 0 and dispersion (covariance) matrix *D=diag(φ_1_,φ_2_)*.

The model is formulated as follows:


$$L_{i}=X_{i}\beta +I_{2}u_{i},i=1,\ldots ,I,$$ (3)

where

*L_i_=(l_i1_,l_i2_*)' is a 2×1 vector and $l_{ik}=logit(p_{ik})=ln\frac{p_{ik}}{1\text{-(}p_{i1}+p_{i2})}$

*k*=1,2; *X_i_*=diag(*X_i1_*,*X_i2_*) is the 2×*m* matrix of auxiliary variables, *m=m_1_+m_2_* where *m_k_* is the number of explanatory variables for category *k*, and *X_ik_=(x_ik1_,x_ik2_,… ,x_ikmk_)* is the set of observations corresponding to area i and category *k, k=1, 2; β=(β_1_,β_2_)* is the *m*×1 regression parameter vector; *I_2_* is the 2×2 identity matrix; and *u_i_=(u_i1_^,^u_i2_)*' is the 2×1 vector of random effects. The logit of a proportion *p* is the log-transformed odds of p, that is *ln(p/1-p).*

Since the model assumes a linear relationship between the explanatory variables and the logit transformation of the prevalence of smokers and ex-smokers, the association between them was assessed in the exploratory analysis using the Spearman correlation coefficient. This coefficient measures the force of the linear relationship between two ordinal or continuous variables. It has the same interpretation as the Pearson correlation coefficient: a value equal to 0 is indicative of no linear relationship, and a value of ± 1 indicates a positive or negative perfect relationship between the variables.

As a result of fitting the model, in which explanatory variables with p<0.05 were maintained, we obtained the estimated prevalence of smokers and ex-smokers in 174 areas used for estimation purposes and then deduced the prevalence of never smokers from these. The prevalence in the six remaining areas was estimated based on the model’s coefficients, using data from the auxiliary variables. The prevalence in the six remaining areas was calculated using the synthetic part of the linear predictor *L_i_=X_i_β*.

To assess the precision of the estimates, we calculated the mean squared error (MSE) using a parametric bootstrap procedure^16^, and on the basis of this we obtained the 95% confidence intervals for the prevalence ($95\%\;\text{CI}:\;\hat{p}\;\pm \;1.96\hat{p}\sqrt{MSE}$) and the coefficients of variation ($\text{CV=}\sqrt{MSE}$). With regard to precision, CV lower than 30% was deemed acceptable, taking into account the criteria applied by the National Center for Health Statistics^2-17^.

To assess the bias of the estimates, we calculated the prevalence derived from the model aggregated by AR, and then compared these against those obtained from the 2017 SNHS with the direct estimator; we conducted the same comparison with prevalence broken down by sex and age group.

Data were processed using the Stata IC v17 software, and the estimation of the model was performed with the MME package for R^18^.

## RESULTS

The sample sizes of the 2017 SNHS in the 180 groups or areas defined for this study were generally small. Hence, the quartiles were Q_1_=73, Q_2_=101 and Q_3_=161, with a range 19–530; the minimum sample size was observed among men aged 65–74 years in Ceuta and Melilla, and the maximum sample size among women aged 35–54 years in Andalusia.

The Spearman correlation coefficients between the explanatory variables and the logit transformation of the prevalence of smokers and ex-smokers, calculated at an area level, ranged 0–0.793 in terms of absolute value (Table 1). The variables showing the closest correlation with the prevalence of smokers were nationality, education level, relationship with activity, and morbidity. In the case of ex-smokers, the most closely correlated variables were the relationship with activity, occupational sector, and morbidity. Table 1 also shows the median and range of values for each variable.

**Table 1:** Median and range of values of the explanatory variables of the model, and Spearman correlation coefficient between each variable and the logit transformation of the prevalence of smokers and ex-smokers

	*Values of the variables*		*Spearman correlation coefficient*	
	*Median %*	*Range %*	*Smokers*	*Ex-smokers*
**Nationality**				
Foreign population	6.42	0.30–23.63	0.579	-0.299
**Degree of urbanization**				
Living in DPA towns	49.11	18.11–100	-0.012	-0.090
Living in IPA towns	31.81	0.00–48.49	0.188	-0.057
Living in TPA towns	16.42	0.00–52.25	-0.084	0.141
**Coastal areas**				
Living in coastal towns	38.90	0.00–100	-0.005	-0.110
Living in inland towns	61.10	0.00–100	0.005	0.110
**Education level**				
Basic education	27.02	5.56–89.24	-0.740	0.240
Secondary education	56.60	8.78–74.69	0.793	-0.262
Higher education	14.50	1.98–37.27	0.700	-0.101
**Relationship with activity**				
Employed	52.84	28.52–88.11	0.433	0.472
Unemployed	10.39	3.59–23.91	0.363	-0.275
Economically inactive	34.49	4.33–59.29	-0.570	-0.328
Employment rate	85.03	59.49–94.64	-0.110	0.401
**Professional category**				
Managers, directors and technicians	31.35	15.42–50.65	-0.195	0.207
Skilled workers	47.33	32.92–61.46	0.040	0.043
Unskilled workers	20.96	8.03–34.38	0.068	-0.208
**Occupational sector with paid employment**				
Industry	8.46	0.59–33.93	0.258	0.384
Construction	6.05	0.10–28.73	0.277	0.557
Services	81.28	46.14–98.03	-0.264	-0.502
**Income level**				
Mean income	11.46	9.18–14.71	-0.027	0.078
Living in sections with DI<P10	0.00	0.00–10.34	0.008	0.143
Living in sections with DI>P90	0.12	0.00–31.11	-0.023	0.039
**Morbidity**				
Lung cancer hospital admissions	0.17	0.00–1.69	-0.464	0.753
COPD hospital admissions	0.66	0.00–13.81	-0.589	0.654

The estimated coefficients of the SAE models fitted for S and ExS are shown in Table 2. All blocks of auxiliary variables, except nationality, contributed significant variables to the ExS model. In the case of S, nationality, degree of urbanization, and occupational sector were excluded from the model. In both models, squared variables, whether or not accompanied by the original variable, proved significant, indicating a non-linear relationship. In the S and ExS models, the variables that contributed most to predicting prevalence were the percentage of hospital admissions due to lung cancer and mean per capita income, in both cases, along with their quadratic form in the model.

**Table 2:** Estimated coefficients of the small-area model (β), standard error (SE), 95% confidence intervals (95% CI), and significance (p)

	*β*	*Exp(β)*	*95% CI*		*p*
**Smokers**					
Constant	2.430		-0.448	5.308	0.098
% inland towns	-0.004	0.996	0.992	1.000	0.032
(% inland towns)^2^	<0.001	1.000	1.000	1.000	0.006
(% basic education)^2^	-0.001	0.999	0.999	1.000	<0.001
(% employed with pay)^2^	0.000	1.000	1.000	1.000	<0.001
% unemployed	0.119	1.127	1.081	1.174	<0.001
(% unemployed)^2^	-0.005	0.995	0.994	0.997	<0.001
% unskilled workers	0.019	1.019	1.011	1.027	<0.001
% lung cancer hospital admissions	2.926	18.661	11.978	29.073	<0.001
(% lung cancer hospital admissions)^2^	-1.447	0.235	0.165	0.335	<0.001
(% COPD hospital admissions)^2^	0.004	1.004	1.001	1.006	0.014
Mean income	-0.728	0.483	0.308	0.757	0.001
(Mean income)^2^	0.029	1.029	1.010	1.049	0.002
% DI<P10	-0.033	0.968	0.948	0.988	0.002
(% DI>P90)^2^	0.001	1.001	1.001	1.001	<0.001
**Ex-smokers**			0.000	0.000	<0.001
Constant	19.712		16.300	23.123	<0.001
% TPA	0.027	1.028	1.022	1.034	<0.001
(% inland towns)^2^	<0.001	1.000	1.000	1.000	<0.001
(% basic education)^2^	-0.001	0.999	0.999	0.999	<0.001
% secondary education	-0.045	0.956	0.943	0.970	<0.001
% employed with pay	0.016	1.016	1.012	1.021	<0.001
(% unemployed)^2^	-0.002	0.998	0.998	0.999	<0.001
% skilled workers	-0.213	0.808	0.735	0.889	<0.001
(% skilled workers)^2^	0.002	1.002	1.001	1.003	<0.001
% unskilled workers	0.133	1.142	1.088	1.198	<0.001
(% unskilled workers)^2^	-0.003	0.997	0.996	0.998	<0.001
% employed with pay in construction	0.046	1.047	1.024	1.070	<0.001
(% employed with pay in construction)^2^	-0.002	0.998	0.998	0.999	<0.001
% lung cancer hospital admissions	3.794	44.450	27.926	70.751	<0.001
(% lung cancer hospital admissions)^2^	-2.016	0.133	0.099	0.179	<0.001
% COPD hospital admissions	0.137	1.147	1.110	1.185	<0.001
Mean income	-2.562	0.077	0.050	0.118	<0.001
(Mean income)^2^	0.104	1.110	1.090	1.129	<0.001

Superscript 2 refers to the square of the variable.

Standardized residuals of the model have been calculated (data not shown) to diagnose the model and test the assumption of linearity between the logit and the independent variables. The residuals of both S and ExS had a mean 0 and variance 1, a symmetric distribution, and the curve was close to normal, though in the case of ExS it displayed higher kurtosis. In summary, the model is adequate and meets the linearity hypothesis. The AIC of the model was 1823.9.

**Figure 1:**
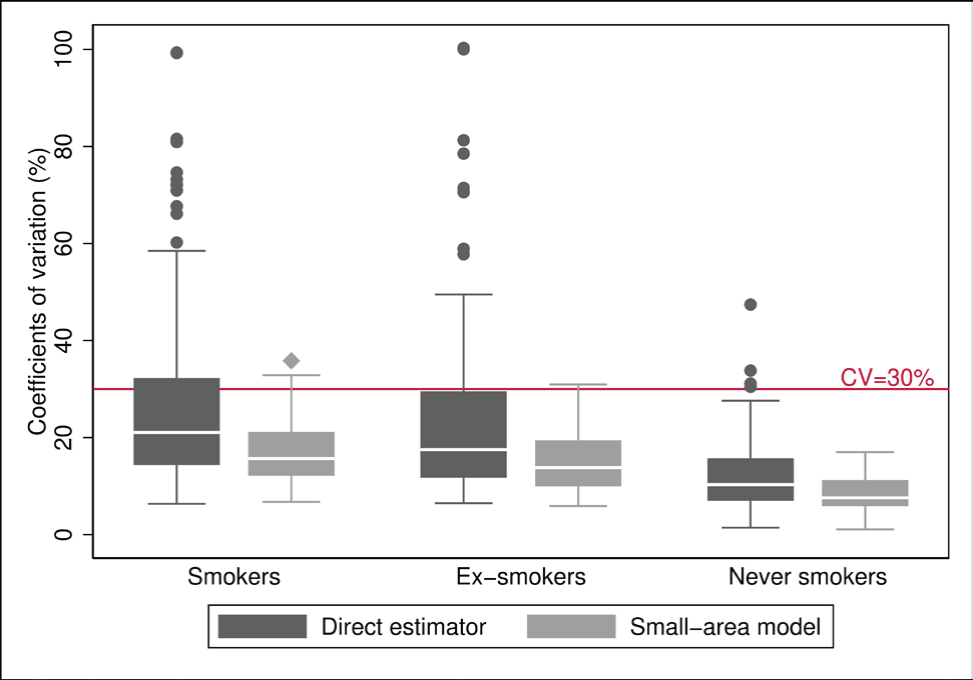
Distribution of the coefficients of variation (%) of the estimators, both direct and based on the small-area model, for prevalences of smokers, ex-smokers, and never smokers: 2017

Concerning the precision of the model, the prevalence estimates of S, ExS and NS obtained from the small-area model had CV lower than 30%, except for seven areas, six in the case of S (with CV ranging 30.9–35.8%) and three areas in the case of ExS (38.8%, 30.9% and 32.0%), with two of the areas being common to S and ExS. Of the seven areas, five corresponded to women and two to men, all from the age group of ≥75 years except one aged 65–74 years. Comparison between model-based CV and direct estimators (Figure 1) showed that better results were obtained in all cases and that the model improved the precision of the estimated prevalence of smokers and ex-smokers. Hence, the median of the CV decreased by 24% for S (21.1% to 15.9%) and 20% for ExS (17.5% to 14.0%), and the 75th percentile decreased by 34% in both cases, going from 32.3% to 21.3% in S and 29.7% to 19.6% in ExS, and the interquartile range was halved, going from 18 to 9, in both cases. Furthermore, the seven areas that maintained a CV above 30% had CV higher than 70% with the direct estimator.

The prevalence estimated with the small-area model and those obtained with the direct estimator at an AR level (54 values) and by sex and age group (54 values), are shown in Supplementary file Tables S2 and S3, respectively, and the distribution of their differences can be seen in Supplementary file Figure S1. As the dotted lines indicate, 94% of the differences were less than 1.5 percentage points in terms of absolute value. If we consider the results by AR there were 6 values outside this interval, one in S, two in ExS, and three in NS, and the greatest differences corresponded to the prevalence of ExS and NS in Cantabria (2.4 and -2.7). According to the 2017 SNHS, in this AR there were 16.9% (95% CI: 14.0–19.8) of ExS and 58.3% (95% CI: 54.2–62.5) of NS, and the model estimated 19.4% (95% CI: 17.3–21.4) and 55.6% (95% CI: 52.7–58.5), respectively. The breakdown by age group and sex showed only one difference greater than 1.5, i.e. among male ex-smokers aged ≥75 years, with a prevalence of 57.9% (95% CI: 54.7–61.1) according to the 2017 SNHS and 59.5% (56.6–62.3) according to the model (difference of 1.6%). Differences with the median closest to 0 were those for smokers by sex and age group (P50= -0.05), and were also those with the least variability, with an interquartile range of 0.73 and a range of -0.6 to 1.0.

**Figure 2:**
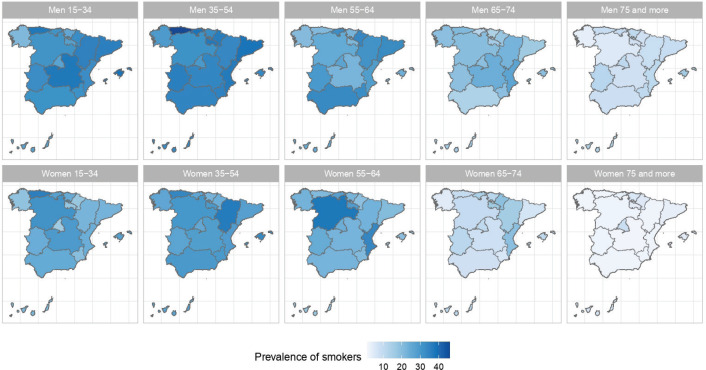
Prevalences of smokers in the ARs of Spain, by sex and age group: 2017

**Figure 3:**
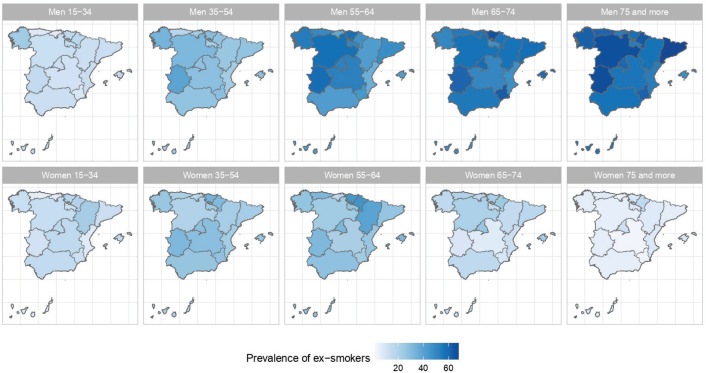
Prevalences of ex-smokers in the ARs of Spain, by sex and age group: 2017

**Figure 4:**
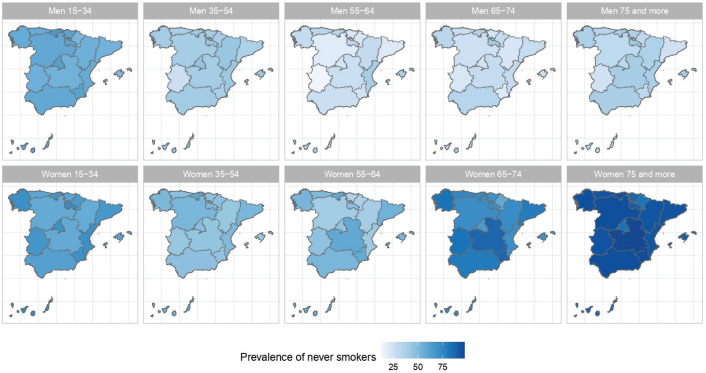
Prevalences of never smokers in the ARs of Spain, by sex and age group: 2017

The results of the small-area model highlight the fact that in Spain there are geographical differences in the prevalence of S, ExS, and NS across all age groups, both in men and women. Figures 2, 3 and 4 show the geographical distribution of the prevalence of S, ExS, and NS, respectively, in each combination of sex and age group. In general, the prevalence of S decreased with age and was lower in women, being the greatest differences in prevalence between ARs, both in males and females, in the age group 55–64 years. The prevalence of ExS rose with age in men, a pattern that was not as clear in women. The prevalence of NS among women aged ≥75 years exceeded 75% in all except two of the ARs. Differences in the prevalence of ex-smokers and never smokers by sex stand out from the age group 55–64 years onwards in all ARs.

## DISCUSSION

This study applied a small-area estimation method to estimate the prevalence of tobacco use in 180 groups in the adult Spanish population, defined based on ARs, sex and age group, clearly improving the precision of the estimates obtained using the direct estimator. The proposed methodology obtained more reliable smoking estimates, in terms of precision and bias, in 180 areas not considered in the SNHS sampling design. The greater part of the CV was lower than 30%, deemed an acceptable cut-off point according to the National Center for Health Statistics standard practice^17^. Regarding bias, deviations between the direct estimates provided by the 2017 SNHS and those based on the SAE model were seen to be <1.5 percentage points in absolute value in 94% of cases. These results indicate that, from a practical point of view, this model should perhaps be automatically applied to SNHS results to show estimates at a subnational level by sex and age. The smoking prevalence by age group and sex at a geographical scale appears to be valid.

Based on the application of this model, tobacco use prevalence in Spain could, for the first time, be ascertained by sex for five age groups in the ARs and Autonomous Cities, thereby avoiding the imprecision of direct estimates obtained after analysis of SNHS data^7^.

SAE methods have been widely used to estimate the prevalence of health-related behaviors or health states at a subnational level, though methods vary from one study to another^2,4,6,19-21^. The same model has been applied to estimate indicators of the relationship with economic activity, such as the proportion of employed and unemployed people and the unemployment rate^6^. Although other studies, such as that of Srebotnjak et al.^6^, chose to fit independent models for each sex, in our case, preliminary analyses showed that the fit of the model and precision of the results were better when both sexes were jointly included.

Some studies which compare different methods^22,23^ conclude that those based on regression models provide more accurate estimates than the synthetic method or spatial smoothing. In the case of tobacco use, various studies have applied multilevel regression models to estimate the prevalence of smokers, introducing covariates at both the individual level and subnational level^2,3,24-26^. These models predict the probability of an individual having a behavior of interest, smoking in this instance, taking into consideration auxiliary covariates, and transforming the probability into prevalence at an area level. As we do not have data on all the individual-level covariates of interest, we resorted to a model at an area level. Our method has the advantage of incorporating the joint distribution of the variable ‘relationship with tobacco use’, categorized into S, ExS, and NS, which allows us to use data from different sources.

Moreover, the multinomial models are optimal for these area-level data since the variables of interest are binary at the unit level, and they are the sum of binary variables at the area level. Also, the totals of S, ExS and NS sum-up the total of the population under study. Therefore, multinomial models that jointly estimate the totals of S, ExS and NS automatically fulfil this restriction. This is an appealing property of these models.

The auxiliary variables included in our model are either important drivers of smoking behavior^27^ or, in the case of hospital admissions due to lung cancer, clear consequences of it. However, the suitability of their inclusion in the model should be individually assessed for each country^28^. To a greater or less extent, SAE estimates at a population level or in specific populations (such as pregnant women) considered auxiliary variables similar to those used in our model. The race is a variable that was considered by the majority of studies. Since no information was available, we have used the percentage of the non-Spanish population as a proxy, as did another study^29^.

It is assumed that the auxiliary variables included in the model are data without random error and should thus be sourced from administrative records. In our study, however, we used data sourced from the Labor Force Survey (LFS) since registered unemployment and Social Security affiliation data with the necessary breakdown level were unavailable. It should be borne in mind here that the LFS sample size is large enough to ensure representativeness and very good precision (n=637152)^11^.

### Strengths and limitations

The model has some limitations. First, being an ecologic study, the associations observed may be different at an individual level. Second, on working with aggregate data, the sample size is reduced to the number of geographical units, with the ensuing loss of statistical power. Third, the structure of the correlation between the outcome variables is not flexible, since the distribution is assumed to be multinomial. As a solution to these limitations, future studies should develop a version of the same model applicable to individual data, thereby making it possible to consider the fact that both individual and ecological aspects influence health-related behaviors. Moreover, to overcome the limitations of the correlation structure, a mixed compositional model could be used at an area level, which would enable a more flexible correlation structure to be defined^30^. Another limitation of our method is that we did not consider spatial correlations. In practice is often reasonable to assume that the effect associated with neighboring AR is proportionally correlated to a measure of distance (not necessarily geographical), with correlations decreasing to zero as the distance increases. Such models are common in spatial statistics. An extension of the model used in this study that includes spatial correlations, may be considered in the future. Regarding covariates, it was not always possible to have contemporary covariate data since the source for covariates such as education level was the Census, the last being from 2011. Regarding the results, the inaccuracy in the prevalence for adults aged ≥75 years should be taken into consideration. This would be related to the disbalance between smokers and non-smokers prevalence, with the former very infrequent. One of this study’s main strengths resides in the auxiliary variables needed to fit the model, since most of these are available at an area level in any country with nationwide health surveys.

The improvement in the precision of estimates obtained after the application of the SAE model is extremely important. Assuming simple random sampling, it would be necessary to collect data on over 160000 persons to achieve such precision, i.e. increasing the SNHS sample seven-fold. If the sample were only to be increased in areas in which the CV was higher than 30%, the overall SNHS sample size would increase four-fold. The economic and personal resources required to conduct the health survey would thus be considerably increased.

The smoking prevalence obtained are estimates, and it is thus possible that local survey data could demonstrate a higher or lower actual prevalence. Validation of our estimates with external data (real-world data) was beyond the scope of this study, but is an important next step.

## CONCLUSIONS

The result of this study reflects the differences in the spatial distribution of the prevalence of tobacco smoking by age and sex in the AR in Spain. Additionally, it demonstrates that area smoking prevalence can be estimated with good precision using exclusively variables from administrative records, thereby making it possible to inform public-health tracking by furnishing estimates down to areas by sex and age group. This means that routine publication of estimates at an area level shortly after the completion of a national health survey is feasible and would also enhance the information given to policy makers and the population.

## Data Availability

The data supporting this research are available from the following source: https://www.sanidad.gob.es/estadisticas/microdatos.do
