# Supplementary material for: Small-area models to assess the geographical distribution of tobacco consumption by sex and age in Spain
Source: Tob Induc Dis. 2023 May 18;21:63. doi: 10.18332/tid/162379 (PMC10194049; doi:10.18332/tid/162379)
Supplement: Supplementary file 1 [file TID-21-63-s1.pdf]

Small-area models to assess the geographic distribution of tobacco consumption by sex and age in Spain

Supplementary Material

Table S1 Auxiliary variables data source

Variable	Data-Source	Web site
**Nationality**	2017 Population Register	INEbase
% foreign population		
**Degree of urbanization**	2017 Population Register	INE
% of population living in densely populated areas (DPA)		
% of population living in intermediate populated areas (IPA)	2016 Eurostat Classification of Cities	EUROSTAT
% of population living in thinly populated areas (TPA)		
**Coastal areas**	2017 Population Register	INE
% of population living in coastal towns and cities		
% of population living in inland towns and cities	2016 Eurostat Classification of Cities	EUROSTAT
**Educational level**	2011 Census	INE
% of population with basic education		
% of population with secondary education		
% of population with higher education		
**Relationship with activity**	2017 Labor Force Survey	INEbase
% of employed population		
% of unemployed population		
% of economically inactive population		
Employment rate		
**Main occupation**		
% of directors, managers, technicians and professionals		
% of skilled workers		
% of unskilled workers		
**Occupational sector**		
% of employed population in industry		
% of employed population in construction		
% of employed population in services		
**Income level**	INE Experimental Statistics. 2017 Atlas of household income distribution	Atlas de distribución de rentas de los hogares
Mean per capita income		
% of population living in towns/cities with a DI=< 10th percentile	Working Group on Social Determinants of Health of the	SEE

% of population living in towns/cities with a DI > 90th percentile	Spanish Society of Epidemiology	
**Morbidity**	Ministry of Health, Consumer Affairs and Social Welfare. General Subdirectorate of Health Information. Registry of Specialized Care Activity -RAE-CMBD of the year 2017	Portal Estadístico del Sistema Nacional de Salud
% of hospitalized population due to lung cancer		
% of hospitalized population due to chronic obstructive pulmonary disease		

Table S2 Prevalences of smokers, ex-smokers and never smokers obtained with the direct estimator (2017 SNHS) and with the small-area model in the 17 ARs and 2 Autonomous Cities of Ceuta and Melilla, together with their 95% confidence intervals (95%CI): 2017

**Direct estimator**	**Smokers**		**Ex-smokers**		**Never smokers**	
**Autonomous Region**	**%**	**95%CI**	**%**	**95%CI**	**%**	**95%CI**
Andalusia	25.3	23.6 - 27.1	24.1	22.4 - 25.8	50.6	48.6 - 52.6
Aragon	26.2	23.2 - 29.4	26.4	23.5 - 29.4	47.4	44.0 - 50.9
Asturias	27.7	24.4 - 31.3	22.4	19.5 - 25.6	49.9	46.1 - 53.7
Balearic Isles	27.5	24.3 - 31.0	25.0	21.9 - 28.3	47.6	43.8 - 51.3
Canary Islands	23.9	21.1 - 27.0	20.5	17.9 - 23.3	55.6	52.2 - 59.0
Cantabria	24.7	21.2 - 28.6	16.9	14.2 - 20.0	58.3	54.2 - 62.4
Castile & Leon	24.9	22.3 - 27.6	26.5	23.9 - 29.2	48.7	45.6 - 51.7
Castile-La Mancha	27.0	24.1 - 30.1	23.8	21.2 - 26.6	49.2	45.9 - 52.4
Catalonia	24.3	22.4 - 26.3	24.9	23.1 - 26.8	50.8	48.6 - 53.0
Valencian Region	26.8	24.6 - 29.2	20.5	18.5 - 22.6	52.7	50.1 - 55.2
Extremadura	26.1	23.0 - 29.5	28.0	25.0 - 31.3	45.8	42.2 - 49.6
Galicia	18.3	16.1 - 20.8	27.8	25.2 - 30.5	53.9	50.9 - 56.9
Madrid	22.2	20.2 - 24.2	28.3	26.2 - 30.4	49.6	47.2 - 52.0
Murcia	26.0	23.1 - 29.2	22.8	20.1 - 25.8	51.2	47.7 - 54.7
Navarre	23.3	19.9 - 27.0	30.2	26.7 - 34.0	46.5	42.4 - 50.6
Basque Country	22.1	19.8 - 24.5	31.0	28.5 - 33.7	46.9	44.1 - 49.8
La Rioja	23.6	20.3 - 27.3	21.4	18.2 - 25.1	54.9	50.8 - 59.0
Ceuta-Melilla	23.7	19.8 - 28.0	12.3	9.3 - 16.2	64.0	59.1 - 68.7
**Small-area model**	**Smokers**		**Ex-smokers**		**Never smokers**	
**Autonomous Region**	**%**	**95%CI**	**%**	**95%CI**	**%**	**95%CI**
Andalusia	25.2	23.4 - 27.0	24.0	22.5 - 25.6	50.7	48.9 - 52.6
Aragon	26.1	23.8 - 28.4	25.9	23.6 - 28.3	48.0	45.8 - 50.2
Asturias	27.1	24.1 - 30.1	22.8	20.6 - 25.1	50.0	47.9 - 52.2
Balearic Isles	27.3	24.6 - 30.1	25.0	22.4 - 27.6	47.6	45.2 - 50.0
Canary Islands	23.7	21.6 - 25.9	21.2	19.1 - 23.3	55.1	52.6 - 57.5
Cantabria	25.0	22.3 - 27.8	19.4	17.3 - 21.4	55.6	52.7 - 58.5
Castile & Leon	24.1	22.0 - 26.2	27.1	25.1 - 29.1	48.8	46.5 - 51.0
Castile-La Mancha	25.3	22.9 - 27.7	24.5	22.3 - 26.7	50.2	47.9 - 52.6
Catalonia	25.3	23.4 - 27.2	24.6	23.0 - 26.2	50.1	48.5 - 51.8
Valencian Region	27.0	25.1 - 28.9	21.1	19.5 - 22.8	51.9	49.8 - 53.9
Extremadura	24.6	22.2 - 27.1	29.4	26.8 - 32.0	46.0	43.3 - 48.7
Galicia	18.8	17.1 - 20.5	27.9	25.6 - 30.2	53.3	51.2 - 55.5
Madrid	21.9	20.1 - 23.7	26.9	25.0 - 28.8	51.1	49.0 - 53.3

Murcia	25.0	22.7 - 27.4	23.6	21.3 - 25.8	51.4	48.7 - 54.1
Navarre	23.9	21.0 - 26.8	30.1	27.4 - 32.8	46.0	43.1 - 49.0
Basque Country	21.4	19.4 - 23.3	31.4	29.3 - 33.5	47.2	44.9 - 49.6
La Rioja	23.7	21.3 - 26.1	23.1	20.8 - 25.4	53.2	50.3 - 56.1
Ceuta-Melilla	24.1	20.7 - 27.5	12.4	10.5 - 14.3	63.5	60.7 - 66.3

Table S3 Prevalences of smokers, ex-smokers and never smokers obtained with the direct estimator (2017 SNHS) and the small-area model, by sex and age group, together with their 95% confidence intervals (95%CI): 2017

**Direct estimator**	**Smokers**		**Ex-smokers**		**Never smokers**	
**Sex and age**	**%**	**95%CI**	**%**	**95%CI**	**%**	**95%CI**
Men 15-34 years	30.9	28.5 - 33.3	11.8	10.2 - 13.5	57.3	54.7 - 59.8
Men 35-54 years	34.0	32.3 - 35.7	27.5	25.9 - 29.1	38.5	36.7 - 40.2
Men 55-64 years	27.8	25.3 - 30.2	48.2	45.5 - 50.9	24.1	21.7 - 26.4
Men 65-74 years	17.4	15.2 - 19.7	55.6	52.7 - 58.6	27.0	24.4 - 29.6
Men 75 years and over	8.6	6.8 - 10.3	57.9	54.7 - 61.1	33.5	30.5 - 36.6
Men >=15 years	28.2	27.2 - 29.3	32.2	31.2 - 33.3	39.5	38.4 - 40.7
Women 15-34 years	22.9	20.9 - 24.9	10.9	9.4 - 12.4	66.2	63.9 - 68.5
Women 35-54 years	28.3	26.7 - 29.9	23.3	21.8 - 24.8	48.4	46.6 - 50.2
Women 55-64 years	24.1	21.8 - 26.4	28.0	25.5 - 30.5	47.9	45.2 - 50.6
Women 65-74 years	8.7	7.2 - 10.1	16.1	14.0 - 18.1	75.3	72.9 - 77.7
Women 75 years and over	2.1	1.3 - 2.9	6.3	5.1 - 7.5	91.7	90.2 - 93.1
Women >=15 years	20.8	19.9 - 21.7	18.0	17.2 - 18.8	61.2	60.1 - 62.2
All 15-34 years	26.9	25.4 - 28.5	11.4	10.3 - 12.5	61.7	60.0 - 63.4
All 35-54 years	31.2	30.0 - 32.3	25.4	24.3 - 26.5	43.4	42.2 - 44.7
All 55-64 years	25.9	24.2 - 27.6	37.9	36.0 - 39.7	36.2	34.4 - 38.1
All 65-74 years	12.8	11.5 - 14.1	34.8	32.9 - 36.8	52.4	50.3 - 54.4
All 75 years and over	4.6	3.8 - 5.5	26.8	25.0 - 28.6	68.6	66.7 - 70.5
All	24.4	23.7 - 25.1	24.9	24.3 - 25.6	50.7	49.9 - 51.4
**Small-area model**	**Smokers**		**Ex-smokers**		**Never smokers**	
**Sex and age**	**%**	**95%CI**	**%**	**95%CI**	**%**	**95%CI**
Men 15-34 years	30.9	28.6 - 33.2	12.2	10.9 - 13.5	56.9	54.8 - 59.0
Men 35-54 years	34.0	32.3 - 35.8	26.5	25.0 - 28.1	39.4	37.8 - 41.0
Men 55-64 years	27.9	25.7 - 30.1	46.8	44.5 - 49.1	25.3	23.5 - 27.1
Men 65-74 years	18.0	16.2 - 19.8	54.8	52.3 - 57.2	27.3	25.3 - 29.3
Men 75 years and over	8.3	7.2 - 9.5	59.5	56.6 - 62.3	32.2	30.1 - 34.3
Men >=15 years	28.3	27.3 - 29.3	31.6	30.8 - 32.5	40.0	39.1 - 41.0
Women 15-34 years	22.6	20.8 - 24.3	12.3	10.9 - 13.6	65.2	63.0 - 67.3
Women 35-54 years	27.7	26.2 - 29.2	24.5	22.9 - 26.1	47.8	46.2 - 49.4
Women 55-64 years	23.8	21.9 - 25.7	28.2	26.2 - 30.3	47.9	46.0 - 49.9
Women 65-74 years	9.5	8.3 - 10.6	16.2	14.7 - 17.8	74.3	72.4 - 76.2
Women 75 years and over	3.1	2.5 - 3.7	6.2	5.4 - 6.9	90.7	89.8 - 91.7
Women >=15 years	20.6	19.8 - 21.3	18.7	17.9 - 19.5	60.7	59.8 - 61.6
All 15-34 years	26.8	25.4 - 28.2	12.2	11.3 - 13.2	61.0	59.5 - 62.5
All 35-54 years	30.9	29.8 - 32.0	25.5	24.4 - 26.6	43.6	42.4 - 44.7
All 55-64 years	25.8	24.3 - 27.3	37.3	35.8 - 38.9	36.9	35.6 - 38.2

All 65-74 years	13.5	12.4 - 14.5	34.3	32.9 - 35.7	52.3	50.9 - 53.6
All 75 years and over	5.2	4.6 - 5.7	27.0	25.8 - 28.2	67.8	66.8 - 68.9
All	24.3	23.7 - 24.9	25.0	24.4 - 25.6	50.7	50.0 - 51.3

Figure S1 Distribution of the differences between estimates, both direct and based on the small-area model, for prevalences of smokers, ex-smokers and never smokers, by AR, and by sex and age group: 2017.

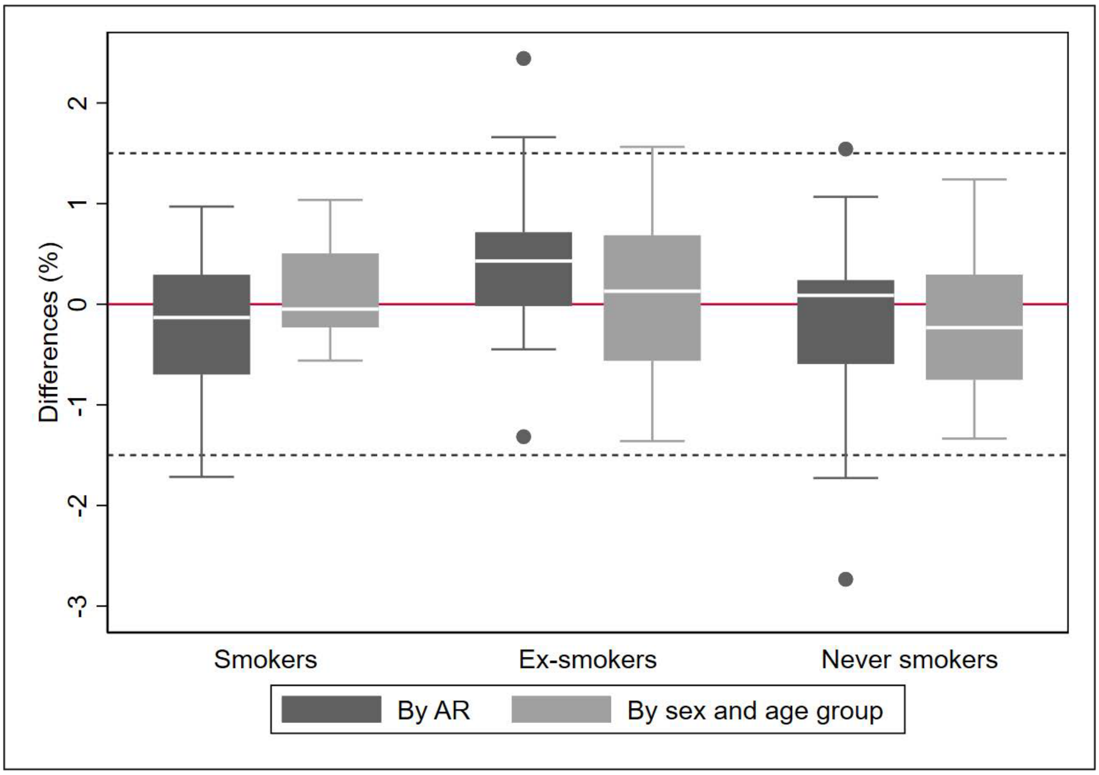

© 2023 Santiago-Pérez M. et al.
